# Impact of neonatal sepsis on serum selenium levels: Evidence of decreased selenium in sepsis-affected neonates

**DOI:** 10.1017/cts.2024.611

**Published:** 2024-10-31

**Authors:** Seyed Hossein Saadat, Rakhshaneh Goodarzi, Zeynab Elahi, Aref Ameri

**Affiliations:** 1 Department of Neonatology, Clinical Research Development Center of Children’s Hospital, Hormozgan University of Medical Sciences, Bandar Abbas, Iran; 2 Department of Pediatrics, Clinical Research Development Center of Children’s Hospital, Hormozgan University of Medical Sciences, Bandar Abbas, Iran

**Keywords:** Neonatal sepsis, selenium, sepsis, systemic inflammatory response syndrome, fever

## Abstract

**Introduction:**

Essential trace elements and micronutrients are critical in eliciting an effective immune response to combat sepsis, with selenium being particularly noteworthy. The objective of this investigation is to analyze and the levels of serum selenium in neonates within sepsis and control groups.

**Methodology:**

In 2023, a case–control study was carried out involving 66 hospitalized infants – 33 diagnosed with sepsis forming the case group and 33 free from sepsis constituting the control group – along with their mothers, at Children’s and Shariati Hospitals in Bandar Abbas. The serum selenium concentrations (expressed in micrograms per deciliter) were quantified utilizing atomic absorption spectrometry. Subsequently, the data were processed and analyzed using IBM SPSS statistical software, version 22.

**Results:**

The average serum selenium level in neonates with sepsis (42.06 ± 20.40 µg/dL) was notably lower compared to the control group (55.61 ± 20.33 µg/dL), a difference that was statistically significant (*p*-value = 0.009). The levels of serum selenium were comparable between neonates and mothers across both study groups.

**Conclusion:**

The findings of this research indicate that selenium levels in the sepsis group were reduced compared to the control group, despite similar selenium levels in the mothers and neonates in both groups, suggesting that sepsis could be associated with a decrease in selenium levels.

## Introduction

Sepsis, a systemic illness resulting from the entry of bacteria, viruses, or fungi into the bloodstream, significantly disrupts the body’s normal hemodynamics [1]. Annually, sepsis accounts for a substantial proportion of mortality in the neonatal age group [2]. Trace elements and micronutrients play a crucial role in mounting an appropriate immune response against sepsis, among which selenium is pivotal [3].

Annually, approximately 2.5 million neonatal deaths are recorded, with a majority of these occurring in low- and middle-income countries [4]. Even with significant progress in neonatal healthcare in developed nations, the worldwide incidence of neonatal sepsis has shown an upward trend. The number of reported cases escalated from 5.59 million in 1990 to 6.31 million in 2019, indicating a growing global challenge in managing this condition [5].

Neonatal sepsis is a systemic event caused by bacteria, viruses, and fungi, accompanied by hemodynamic changes and other clinical findings, leading to mortality. Generally, sepsis involves the presence of infection and the systemic inflammatory response syndrome, which includes respiratory instability (tachypnea or bradypnea), cardiac instability (tachycardia or bradycardia), body temperature variations (fever or hypothermia), and changes in white blood cell count (leukocytosis or leukopenia) [6]. Early-onset neonatal sepsis is defined as the manifestation of sepsis symptoms from birth up to 72 hours (three days) postpartum. This form of sepsis, frequently attributed to infections like Group B Streptococcus, is generally acquired either prenatally or during the birthing process. In contrast, late-onset neonatal sepsis is characterized by the onset of sepsis symptoms after 72 hours and up to the end of 28 days postpartum. This later occurrence of sepsis is often associated with exposure to pathogens prevalent in hospital or community environments [7].

Prematurity and low birth weight are significant risk factors for neonatal infection. Risk factors for early-onset neonatal sepsis include maternal colonization with Group B Streptococcus, chorioamnionitis, premature rupture of membranes, prolonged rupture of membranes (over 18 hours), preterm labor (less than 37 weeks), and multiple pregnancies [8]. Risk factors for late-onset neonatal sepsis include prematurity, low birth weight, prolonged use of indwelling catheters, invasive procedures, ventilator-associated pneumonia, and prolonged antibiotic use [9]. Maternal urinary tract infection and endotracheal intubation during resuscitation at birth are other risk factors for neonatal infection.

Micronutrients, a group of organic vitamins and trace elements, play a vital role in a wide range of essential bodily functions and homeostasis. They act as cofactors for various metabolic enzymes, regulate gene transcription, and bolster the body’s defense against oxidative stress. Micronutrients play a significant role in immune response through their antioxidant activities and regulation of cytokine production. Antioxidant enzymes such as copper, zinc, and manganese superoxide dismutase require trace elements for their biological activities [10].

Selenium (Se) is a vital trace element for human health, integral to several metabolic pathways involved in antioxidant defense and immune system function in mitochondria [11]. During the third trimester of pregnancy, selenium is transferred to the neonate through the placenta and gradually accumulates in the liver from weeks 20 to 40 of pregnancy. Selenium levels are believed to have a direct correlation with gestational age, birth weight, and the Apgar score at 5 minutes [12]. Selenium deficiency increases the production of reactive oxygen species, diminishes the killing capacity of neutrophils, the number of *T* cells and IL-2R affinity, and introduces *T* cells, proliferation and differentiation of *T* cells, lymphocyte toxicity, NK cell activity, and decreases IgG and IgM levels and antibody response. Selenium deficiency reduces the levels of IgM and IgG and compromises immunity, increasing the risk of infection and potentially cancer [13]. The function of innate immune cells may also be affected by selenium levels. Macrophages, through their inflammatory messaging and antipathogen activities, can be influenced by selenium levels [14].

Documented effects of selenium deficiency in neonates include sepsis, low birth weight (LBW), gestational diabetes, preterm labor, and diaphragmatic hernia. In adults, reduced selenium levels have been linked to sepsis, multi-organ failure, and Keshan disease. Selenium deficiency has been observed in patients admitted to neonatal intensive care units (NICU) and Pediatric Intensive Care Units (PICU), not solely due to sepsis, but without a definitive cause identified.

While various factors predispose neonates to infection, many cases occur in the absence of known infection risk factors. Given the impact of trace elements on the immune system, there is a likelihood of increased sepsis risk in patients with a deficiency of these elements, both in the presence and absence of known infection risk factors. Considering the limited studies conducted, often on animal models or adults without sepsis (critically ill) or with sepsis, and the few studies on the role of the micronutrient selenium in neonatal sepsis, this study was designed to determine and compare serum selenium levels in neonates with sepsis versus those without sepsis admitted to Bandar Abbas Children’s and Shariati Hospitals during 2022.

## Methodology

### Study design and setting

This study, structured as a case–control investigation, aims to evaluate serum selenium levels in the context of neonatal sepsis. The research was meticulously conducted at two critical healthcare institutions: Bandar Abbas Children’s Hospital and Shariati Hospital. The duration of the study encompassed the entire year of 2022. These hospitals, known for their comprehensive neonatal care, provided a robust and relevant setting for assessing the correlation between selenium levels and neonatal sepsis.

### Population and sample

The case group consisted of neonates with a gestational age of 37 complete weeks or more and diagnosed with neonatal sepsis, admitted to the neonatal ward or NICU of the Children’s Hospital. The control group included neonates with a gestational age of 37 complete weeks or more, without signs of neonatal sepsis, admitted for reasons other than sepsis. Mothers of neonates in both groups were also included in the study.

### Study entry criteria

- Neonates with a gestational age of 37 complete weeks or more (both case and control groups).
- Age from 0 hours to 28 days (both case and control groups).
- Presence of clinical and laboratory signs of sepsis in the case group.
- Absence of clinical signs of sepsis in the control group.

### Exclusion criteria

- Presence of evident congenital anomalies.
- Onset of sepsis symptoms in the control group.
- Resuscitation and asphyxia at birth.
- Indicators suggesting chromosomal disorders.
- Presence of risk factors for early- and late-onset sepsis such as chorioamnionitis, premature rupture of membranes, prolonged rupture of membranes (more than 18 hours), preterm labor (less than 37 weeks), etc.
- Use of medications known to affect selenium levels.

### Sampling method and data collection

A convenience sampling approach was used. The study included 66 neonates with a gestational age of 37 complete weeks or more and their mothers, aged from 0 hours to 28 days post-birth. Neonates meeting the clinical and laboratory criteria for sepsis were included in the case group. Neonates without clinical signs of sepsis were chosen as the control group. A 2.5 cc blood sample was collected from neonates at the time of hospitalization in both groups to determine serum selenium levels and sent to the laboratory for analysis.

### Criteria for selecting neonates with sepsis

At least two of the following sepsis-related symptoms:

- Clinical variables:
  - Temperature dysregulation (central temperature > 38.5°C or < 36°C).
  - Heart rate ≥ 180 beats per minute or ≤ 100 beats per minute.
  - Respiratory rate > 60 breaths per minute with reduced arterial oxygen saturation.
  - Ventilation insufficiency (need for respiratory support including oxygen hood, Continuous Positive Airway Pressure [CPAP], and ventilator).
  - Lethargy or altered level of consciousness.
  - Hypotonia.
  - Feeding intolerance or difficulties.
  - Glucose intolerance (blood sugar >180 mg/dL or < 40 mg/dL).
- Hemodynamic variables:
  - Systolic blood pressure <50 mm Hg on the first day of birth.
  - Systolic blood pressure <65 mm Hg after the first day.
  - Capillary refill time >3 s.
- Accompanied by:
  - CRP ≥ 10 mg/dL.

### Data Collection and measurement of serum selenium levels

Selenium levels were measured using the atomic absorption method. Collected data included age, sex, birth weight, gestational age, serum selenium levels, type of delivery, complete blood count (CBC) findings, C-Reactive Protein (CRP), type of antibiotic used and the need for antibiotic change, blood culture, and duration of hospitalization.

### Study design and data analysis method

Data were analyzed using IBM SPSS statistical software, employing descriptive methods such as tables and statistical indices such as mean, standard deviation, and frequency. Quantitative data were analyzed using *t*-tests, and qualitative data with chi-square tests. Logistic regression was used to calculate the relative risk of developing sepsis based on the results obtained. A *p*-value of <0.05 was considered statistically significant.

## Results

A total of 66 patients were included in the study: 33 patients with sepsis as the case group and 33 patients without sepsis as the control group, all meeting the study’s entry criteria.

Table 1 presents a comprehensive analysis of the demographic information of the neonates involved in the study. It delineates key variables such as age at admission, birth weight, and gestational age.

**Table 1.** Demographic information of neonates

Variable	Group	Mean	Standard deviation	*p*-value
Age at admission (Days)	Control	6.03	2.54	0.515
	Sepsis	2.87	4.42	
Birth weight (Grams)	Control	3096.67	378.83	0.434
	Sepsis	3019.09	419.77	
Gestational age (Weeks)	Control	38.42	0.71	0.601
	Sepsis	38.55	1.12	

Table 2 provides a detailed overview of gender distribution within the study’s cohorts. It distinctly categorizes the neonates based on gender (male and female) across both the control and sepsis groups, comparing the number and percentage of each gender within these groups using Chi-square test. A noteworthy observation from the table is the slight variance in gender prevalence between the groups.

**Table 2.** Gender distribution in both groups

Group	Gender	Frequency (Percentage)	*p*-value
Control	Male	18 (58%)	0.617
	Female	15 (42%)	
Sepsis	Male	21 (62%)	
	Female	12 (38%)	

Table 3 provides a comparative analysis of serum selenium concentrations in neonates, segmented into two groups: one with sepsis and the other serving as a control. The analysis reveals that the mean serum selenium level in the sepsis group is 42.06 µg/dL, while the control group displays a higher mean level at 55.61 µg/dL (*p*-value = 0.009).

**Table 3.** Comparison of serum selenium levels (µg/dL)

Variable	Group	Mean	Standard deviation	*p*-value
Neonates Serum Selenium Level	Sepsis	42.06	20.40	0.009
	Control	55.61	20.33	

**Figure 1.**
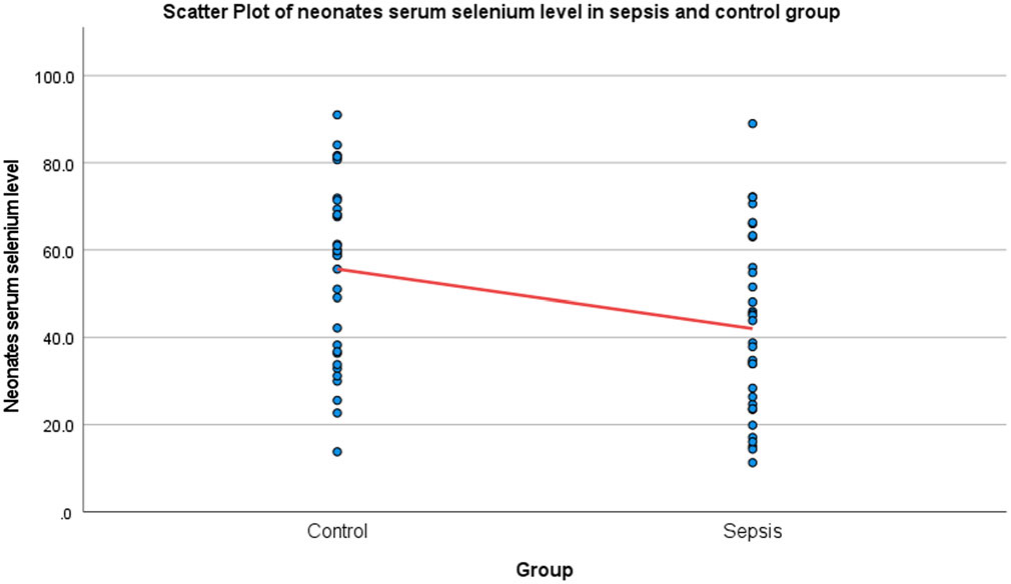
Scatter plot of neonates serum selenium level in sepsis and control group. Table 4 provides a comparison of serum selenium levels between neonates and their mothers in both sepsis and control groups. In the sepsis group, neonates have a mean serum selenium level of 42.06 µg/dL, while mothers show a slightly higher level of 45.39 µg/dL, with a *p*-value of 0.973 indicating no significant difference between the two. In the control group, neonates exhibit a mean level of 55.61 µg/dL, compared to mothers who have a mean level of 52.28 µg/dL, with a corresponding *p*-value of 0.207, again suggesting no significant difference between neonates and mothers in serum selenium levels in this group. This concise comparison in Table 4 highlights the similarities in selenium levels across both groups, irrespective of the sepsis condition.

Distribution of serum selenium levels of neonates is shown in Figure 1.

**Table 4.** Comparison of serum selenium level in mothers and neonates in sepsis and control groups

Group	Group	Mean	Standard deviation	*p*-value
Sepsis	Neonatal Serum Selenium Level	42.06	20.40	0.973
	Maternal Serum Selenium Level	45.39	21.73	
Control	Neonatal Serum Selenium Level	55.61	20.33	0.207
	Maternal Serum Selenium Level	52.28	20.68	

Table 4 provides a comparison of serum selenium levels between neonates and their mothers in both sepsis and control groups. In the sepsis group, neonates have a mean serum selenium level of 42.06 µg/dL, while mothers show a slightly higher level of 45.39 µg/dL, with a *p*-value of 0.973 indicating no significant difference between the two. In the control group, neonates exhibit a mean level of 55.61 µg/dL, compared to mothers who have a mean level of 52.28 µg/dL, with a corresponding *p*-value of 0.207, again suggesting no significant difference between neonates and mothers in serum selenium levels in this group. This concise comparison in Table 4 highlights the similarities in selenium levels across both groups, irrespective of the sepsis condition.

Neonatal selenium level was positively correlated with maternal selenium level (Pearson correlation coefficient = 0.560, *p*-value < 0.001). This indicates a statistically significant association, suggesting that higher maternal selenium levels correspond with higher neonatal selenium levels.

Table 5 presents analysis of serum selenium levels categorized by gender within control and sepsis groups. In the control group, there are 18 males with a mean selenium level of 51.47 µg/dL and a standard deviation of 21.98, and 15 females with a higher mean level of 60.57 µg/dL and a standard deviation of 17.60; the p-value for males is 0.205, indicating no significant gender-based difference. In the sepsis group, 21 males have a mean selenium level of 41.03 µg/dL with a standard deviation of 20.36, and 12 females have a mean level of 43.84 µg/dL and a standard deviation of 21.23; the p-value for males is 0.710, again suggesting no significant gender-based difference in selenium levels. This table provides a clear gender-based comparison within each group, highlighting that the differences in serum selenium levels between males and females are not statistically significant, both in control and sepsis conditions.

**Table 5.** Comparison of serum selenium levels (µg/dL) by gender

Group	Gender	Mean	Standard deviation	*p*-value
Control	Male	51.47	21.98	0.205
	Female	60.57	17.60	
Sepsis	Male	41.03	20.36	0.710
	Female	43.84	21.23	

## Discussion

In this study, we aimed to explore the role of selenium in sepsis in neonates. Given the scarcity of research in the neonatal field, we initiated a study to investigate the relationship between selenium and sepsis, hoping to contribute to the treatment of neonatal sepsis.

The primary objective was to compare selenium levels between the sepsis and control groups. We found that the selenium levels in neonates with sepsis were significantly lower than those in the control group. However, no significant difference was found between selenium levels in neonates and mothers in sepsis and control group. This suggests that the reduced selenium levels are a consequence of sepsis in neonatal sepsis.

Evidence from the systematic review conducted by Bhawan Deep Garg et al. [15] suggests that selenium supplementation may play a role in the prevention of late-onset sepsis (LOS) in very low birth weight (VLBW) neonates. This review, encompassing randomized controlled trials, indicates a statistically significant reduction in the incidence of LOS among neonates receiving selenium supplementation. The findings highlight the potential of selenium as an agent with antioxidant and immune-modulating properties in the context of neonatal sepsis. However, it is important to note the difference in the patient population in our study compared to the one reviewed by Garg et al. Our research focused on term neonates, which presents a distinct demographic from the very low birth weight neonates considered in the systematic review. While the results of the review suggest beneficial effects of selenium supplementation in reducing LOS among VLBW neonates, the applicability of these findings to term neonates may not be directly comparable. The variance in birth weights and gestational ages could potentially influence the outcomes and efficacy of selenium supplementation. Therefore, while the review provides valuable insights into the role of selenium in neonatal sepsis, further research is warranted to explore its impact specifically in term neonates.

In their investigation, Julian Hackler and colleagues [16] explored the significance of selenium (Se) and copper (Cu) levels, as well as the biomarkers selenoprotein P (SELENOP) and ceruloplasmin (CP), in identifying early-onset sepsis (EOS) in neonates. The study suggests that changes in the status of Se and Cu, along with variations in SELENOP and CP levels, might serve as potential indicators of EOS.

The study by Ramin Iranpour et al.[17] illustrates a notable difference in serum selenium levels between preterm and term infants. Our research, conducted on term infants, explores the relationship between neonatal sepsis and serum selenium levels, an area that has received limited attention in previous studies. The established distinction in serum selenium levels between preterm and term neonates poses a limitation when comparing our findings with research focused on preterm neonates. This differentiation highlights the importance of considering gestational age in studies examining neonatal selenium levels and their potential health implications.

Considering the research by Kristin Varsi et al. [18] we recognize that low maternal selenium levels are correlated with an increased risk of infection in infants. Their study, focusing on selenium levels in pregnant and lactating women and their infants, demonstrates a link between insufficient maternal selenium during pregnancy and an elevated risk of infant infection in the early stages of life.

The study by Rahul Aggarwal et al. [19] was centered on assessing the impact of selenium supplementation in the prevention of late-onset sepsis among preterm neonates with VLBW. This research is particularly noteworthy for underscoring the antioxidant attributes of selenium and its possible influence in diminishing mortality related to sepsis in neonates.

However, a significant distinction exists between the demographics of our study and that of Aggarwal et al. Their research primarily involved preterm neonates characterized by extremely low birth weights, specifically under 1500 g, and a gestational age under 32 weeks. Our investigation, on the other hand, was exclusively focused on term neonates. This distinction is of paramount importance, as the behavior of selenium and the susceptibility to sepsis in term neonates may significantly diverge from those observed in VLBW preterm neonates. Thus, while Aggarwal et al.’s findings offer crucial insights into the role of selenium in combating neonatal sepsis, the direct correlation of their findings with our study is potentially limited, considering the disparities in gestational age and birth weight.

In the context of Aggarwal et al.’s research, the administration of selenium was found to notably enhance the levels of serum selenium and concurrently lower the occurrence of late-onset sepsis in the studied group. Our research, which delves into the realm of term neonates, contributes a new perspective to the existing knowledge. It investigates serum selenium levels within the scope of neonatal sepsis and supports the hypothesis that selenium could be a key factor in the immune response of neonates, regardless of their birth weight and gestational age.

Regarding gender, our study included 40 male and 26 female neonates, but no significant gender-related difference in selenium levels or infection rates was observed. This indicates that gender does not significantly impact selenium levels or the occurrence of infection in this study. However, we did not conduct a priori power analysis to determine the sample size required to detect a sex-based effect of this magnitude. It is possible that our study was underpowered, and a larger sample size might have revealed a statistically significant difference. Future research with a larger and more representative sample could further explore potential sex-based differences.

Another limitation of our study is that we did not follow the neonates to discharge. This limits our ability to investigate the potential association between the lowest selenium levels at birth and poorer clinical outcomes such as prolonged hospitalization, need for intensive care, or increased risk of complications.

Our study acknowledges several other limitations that could be addressed in future research to strengthen the understanding of the relationship between selenium and neonatal sepsis. Including more detailed clinical data beyond baseline levels, such as the specific types of organisms causing sepsis and the presenting symptoms, could provide a richer understanding of the association between selenium and disease severity. While obtaining extensive data from neonates can be challenging, future studies could aim for a more comprehensive characterization of the sepsis cases.

A more comprehensive and detailed table outlining patient demographics, including factors such as type of feeding (breastmilk vs. formula), could provide valuable context for interpreting baseline selenium levels and potential variations.

Future research could benefit from exploring established clinical thresholds for selenium deficiency and relating them to the observed changes in selenium levels within our study population. This would help clarify the clinical significance of the statistically significant findings.

Detailed clinical course descriptions for individual patients could potentially shed light on additional factors influencing selenium levels. While this study couldn’t delve into specifics due to sample size limitations, future research with larger and more detailed datasets could explore these factors in more depth, potentially revealing new areas of investigation.

For future studies, it is recommended to check selenium levels on the third and fifth days of the illness to better understand the progression and recovery of selenium levels in relation to neonatal health. The recommendation for serial selenium measurements in future studies stems from the potential role of selenium in the immune response during sepsis. We hypothesize that selenium may be consumed by the immune system during the inflammatory response to sepsis, leading to depletion. This raises intriguing questions about selenium’s role in both sepsis susceptibility and disease severity. Further research is needed to explore this hypothesis and investigate if selenium levels differ based on the type of sepsis (bacterial vs. viral). Additionally, studies examining if selenium levels recover after sepsis resolution would be valuable in understanding its dynamic role in the context of infection.

## Conclusion

Serum selenium concentrations in neonates with sepsis were markedly reduced in comparison to the control group. A notable finding of this research is the lack of substantial variation in selenium levels between mothers of neonates in both the sepsis and control groups. This observation supports the theory that sepsis may lead to a decrease in selenium, an essential micronutrient recognized for its antioxidant properties and immune-modulating effects.

This research highlights the necessity for additional investigations to understand how sepsis affects selenium levels and to assess the potential of selenium supplementation as a therapeutic approach in neonatal sepsis management. Furthermore, given the absence of significant differences in selenium levels among mothers, future studies should delve into the dynamics of selenium transmission during gestation and its impact on the immune response and vulnerability to infections like sepsis in neonates.

## References

[1] Odabasi IO , Bulbul A. Neonatal sepsis. Sisli Etfal Hastan Tip Bul. 2020; 54(2): 142–158. 32617051 10.14744/SEMB.2020.00236 PMC7326682

[2] Fleischmann C , Reichert F , Cassini A et al. Global incidence and mortality of neonatal sepsis: a systematic review and meta-analysis. Arch Dis Child. 2021; 106(8): 745–752. 33483376 10.1136/archdischild-2020-320217 PMC8311109

[3] Weyh C , Kruger K , Peeling P , Castell L. The role of minerals in the optimal functioning of the immune system. Nutrients 2022; 14(3): 644. 35277003 10.3390/nu14030644 PMC8840645

[4] Rosa-Mangeret F , Benski AC , Golaz A et al. 2.5 million annual deaths-are neonates in low- and middle-income countries too small to be seen? A bottom-up overview on neonatal morbi-mortality. Trop Med Infect Dis. 2022; 7(5): 64. 35622691 10.3390/tropicalmed7050064 PMC9148074

[5] Li J , Xiang L , Chen X et al. Global, regional, and national burden of neonatal sepsis and other neonatal infections, 1990–2019: findings from the global burden of disease study 2019. Eur J Pediatr. 2023; 182(5): 2335–2343. 36879151 10.1007/s00431-023-04911-7

[6] Hotchkiss RS , Moldawer LL , Opal SM , Reinhart K , Turnbull IR , Vincent JL. Sepsis and septic shock. Nat Rev Dis Primers 2016; 2(1): 16045. 28117397 10.1038/nrdp.2016.45 PMC5538252

[7] Cortese F , Scicchitano P , Gesualdo M et al. Early and late infections in newborns: where do we stand? A review Pediatr Neonatol. 2016; 57(4): 265–273. 26750406 10.1016/j.pedneo.2015.09.007

[8] Adair CE , Kowalsky L , Quon H et al. Risk factors for early-onset group B streptococcal disease in neonates: a population-based case-control study. CMAJ. 2003; 169(3): 198–203. 12900477 PMC167120

[9] Shah BA , Padbury JF. Neonatal sepsis: an old problem with new insights. Virulence 2014; 5(1): 170–178. 24185532 10.4161/viru.26906 PMC3916371

[10] Cunningham-Rundles S , Lin H , Ho-Lin D , Dnistrian A , Cassileth BR , Perlman JM. Role of nutrients in the development of neonatal immune response. Nutr Rev. 2009; 67 Suppl 2(0 2): S152–S163. 19906219 10.1111/j.1753-4887.2009.00236.x PMC4566995

[11] Zhang F , Li X , Wei Y. Selenium and selenoproteins in health. Biomolecules 2023; 13(5): 799. 37238669 10.3390/biom13050799 PMC10216560

[12] Biswas K , McLay J , Campbell FM. Selenium supplementation in pregnancy-maternal and newborn outcomes. J Nutr Metab. 2022; 2022: 4715965. 35571749 10.1155/2022/4715965 PMC9095401

[13] Avery JC , Hoffmann PR. Selenium,selenoproteins, and immunity. Nutrients 2018; 10(9):1203. 30200430 10.3390/nu10091203 PMC6163284

[14] Huang Z , Rose AH , Hoffmann PR. The role of selenium in inflammation and immunity: from molecular mechanisms to therapeutic opportunities. Antioxid Redox Signal. 2012; 16(7): 705–743. 21955027 10.1089/ars.2011.4145 PMC3277928

[15] Garg BD , Bansal A , Kabra NS. Role of selenium supplementation in prevention of late onset sepsis among very low birth weight neonates: a systematic review of randomized controlled trials. J Matern Fetal Neonatal Med. 2019; 32(24): 4159–4165. 29792085 10.1080/14767058.2018.1481039

[16] Hackler J , Wisniewska M , Greifenstein-Wiehe L et al. Copper and selenium status as biomarkers of neonatal infections. J Trace Elem Med Biol. 2020; 58: 126437. 31778962 10.1016/j.jtemb.2019.126437

[17] Iranpour R , Zandian A , Mohammadizadeh M , Mohammadzadeh A , Balali-Mood M , Hajiheydari M. Comparison of maternal and umbilical cord blood selenium levels in term and preterm infants. Zhongguo Dang Dai Er Ke Za Zhi. 2009; 11(7): 513–516. 19650978

[18] Varsi K , Bolann B , Torsvik I , Rosvold Eik TC , Hol PJ , Bjorke-Monsen AL. Impact of maternal selenium status on infant outcome during the First 6 Months of life. Nutrients 2017; 9(5):486. 28492511 10.3390/nu9050486 PMC5452216

[19] Aggarwal R , Gathwala G , Yadav S , Kumar P. Selenium supplementation for prevention of late-onset sepsis in very low birth weight preterm neonates. J Trop Pediatr. 2016; 62(3): 185–193. 26867560 10.1093/tropej/fmv096

